# Long-Term Retrospective Analysis of Microvascular Decompression in Patients With Recurrent Trigeminal Neuralgia

**DOI:** 10.3389/fneur.2020.584224

**Published:** 2020-12-21

**Authors:** Jiayu Liu, Guangyong Wu, Hui Xiang, Ruen Liu, Fang Li, Bo Hei, Weiqiang Qian, Haidong Song, Zhi Liu

**Affiliations:** ^1^Department of Neurosurgery, Peking University People's Hospital, Beijing, China; ^2^Department of Neurosurgery, The Hospital of Shunyi District Beijing, Beijing, China; ^3^Department of Neurosurgery Jiangxi Provincial People's Hospital Affiliated to Nanchang University, Nanchang, China

**Keywords:** trigeminal neuralgia, recurrence, microvascular decompression, nerve combing, surgical outcome

## Abstract

**Objective:** To explore the clinical characteristics of patients with recurrent trigeminal neuralgia (TN) and the experience of microvascular decompression (MVD) in the treatment of such patients.

**Methods:** We retrospectively analyzed clinical data, imaging examination results, surgical methods, and treatment efficacies in 127 patients with recurrent typical TN from January 2005 to December 2014.

**Results:** The age of the recurrent group was higher than that of the non-recurrent group (*p* < 0.05). The duration of pain before the first MVD procedure was longer in the recurrent group than in the non-recurrent group (*p* < 0.05). Patients in the recurrent group were more likely to have compression of the trigeminal nerve by the vertebrobasilar artery (VBA) or multiple vessels than patients in the non-recurrent group (*p* < 0.05). A Kaplan–Meier curve showed a median pain-free survival of 12 months after the first MVD procedure. The severity of pain (preoperative visual analog scale [VAS] score) in patients with recurrence was lower than that in patients with first-onset TN (*p* < 0.05). Vessel compression, Teflon compression or granuloma and arachnoid adhesion were considered the main causes of recurrence. Postoperative Barrow Neurological Institute (BNI) scores in the redo MVD group were excellent (*T* = 2) for 69 patients (53.33%) and good (*T* = 3) for 46 patients (36.22%). The postoperative follow-up was 63–167 months (105.92 ± 25.66). During the follow-up, no recurrence was noted. All complications were cured or improved.

**Conclusions:** Microvascular decompression (MVD) is an effective surgical method for the treatment of TN. For recurrent patients, reoperation can achieve good results.

## Introduction

Trigeminal neuralgia (TN) is a type of paroxysmal, brief, intense pain that occurs repeatedly in the trigeminal nerve region and is divided into two types: typical and atypical. The etiology of TN is unknown. Typical TN is mostly caused by neurovascular conflict (NVC) of the trigeminal nerve (1). The treatment of typical TN by microvascular decompression (MVD) is based on NVC. Peter Janetta started performing MVD in the 1960s, which is recognized as the only treatment that can eliminate the cause (2). The clinical treatment of TN with MVD surgery has a cure rate of 80–98%, but the follow-up recurrence rate is 10–30% (3). The purpose of this study was to explore the clinical characteristics of patients with recurrent TN and the experience of MVD in the treatment of such patients to accumulate additional clinical evidence for optimal treatment protocols.

## Materials and Methods

### Patients

**Figure 1:**
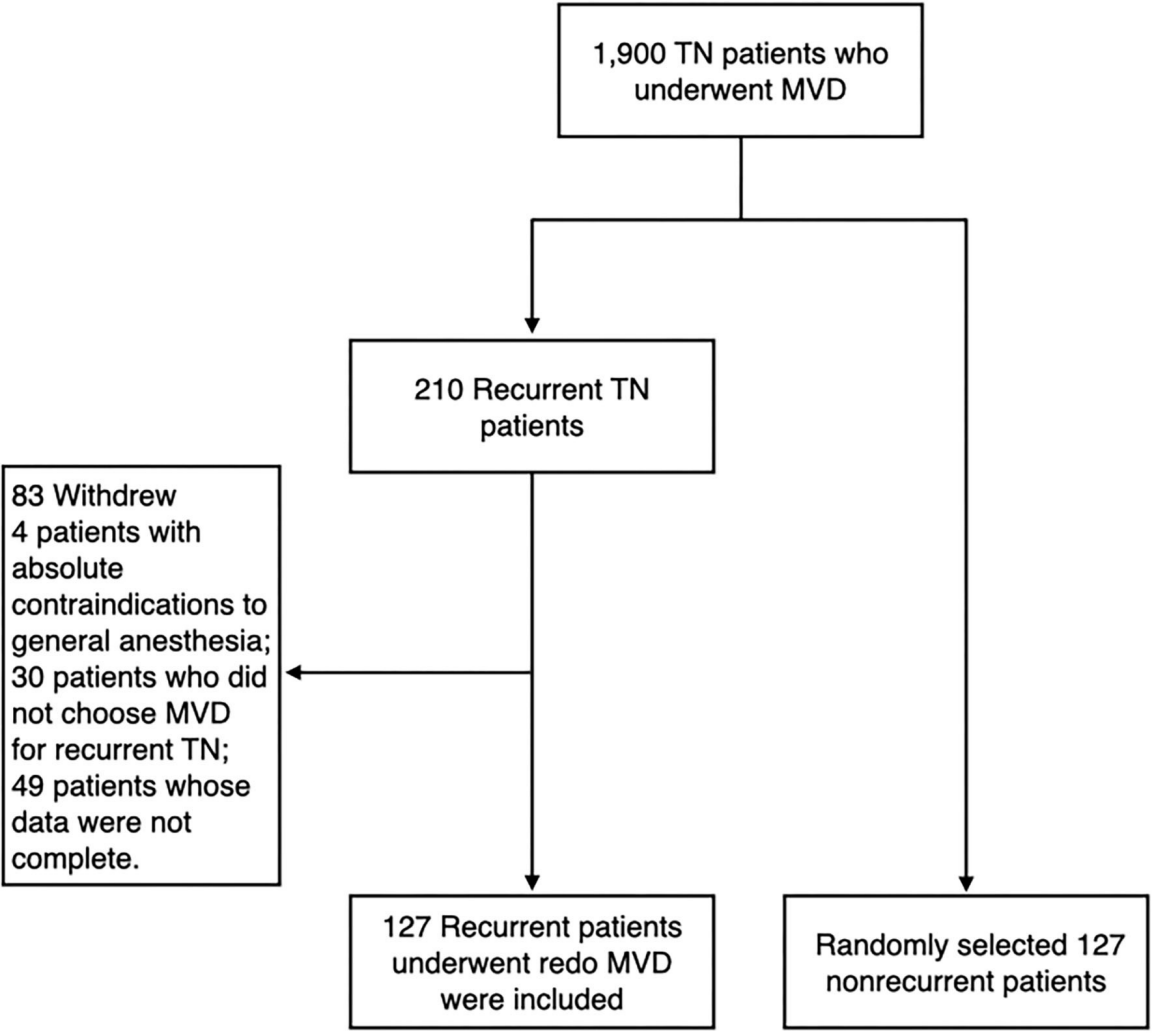
A flowchart of the recruitment.

We continuously followed 1,900 patients with TN who underwent MVD at the Department of Neurosurgery, Jiangxi provincial People's Hospital, the Seventh Medical Center of PLA General Hospital and Characteristic Medical Center of Strategic Support Force from January 2005 to December 2014. Patients with secondary or atypical TN and incomplete clinical data were excluded from the study. Among the followed patients, recurrence developed in 210 patients after initial pain improvement. A total of 127 patients underwent redo MVD. In addition, we randomly selected 127 non-recurrent patients as the control group (Figure 1). The method of random sampling was to use random number generator from SPSS statistical software 19.0 (IBM Corp., Armonk, NY, USA). Operations were carried out by the corresponding author, Ruen Liu. The study was approved by the institutional review board of the hospitals.

The diagnosis was made based on the criteria of the International Classification of Headache Disorders, the 3rd edition, (ICHD-3). Surgical treatment was offered after failure of medical management or percutaneous procedures. MVD was offered if a patient's condition indicated that he or she was fit for general anesthesia regardless of age. Recurrent TN was defined as the resurgence of TN pain on the same side after previous successful MVD with complete pain relief without any medication.

The primary inclusion criteria were typical, drug-resistant TN (the International Headache Society criteria), or drug-responsive TN but with severe drug related adverse effects, without absolute contraindications to general anesthesia (4). The exclusion criteria were as follows: (1) patients with absolute contraindications to general anesthesia; (2) patients who did not choose MVD for recurrent TN; (3) patients whose data were not complete.

### Preoperative Management

A preoperative MRI examination was performed in all patients, including 3D T1- and T2-weighted high-resolution sequences, for clear visualization of the trigeminal nerve and all vascular structures. The use of 3D time-of-flight magnetic resonance angiography (MRA) allowed visualization of only vessels with high flow, which are principally arteries. However, the vascular statistics in the study were based on intraoperative observations.

### Operative Technique

After the induction of general anesthesia, the patient was placed in the lateral park bench position with three-point fixation, and retrosigmoid craniotomy was performed. After opening the dura mater, the cerebellar horizontal fissure was carefully dissected to minimize retraction of the acoustic nerve. With maximum protection of the petrosal veins, the trigeminal nerve was exposed. We inserted a prosthesis between the offending vessels and the affected nerve to separate the nerve-vessel conflict.

**Figure 2:**
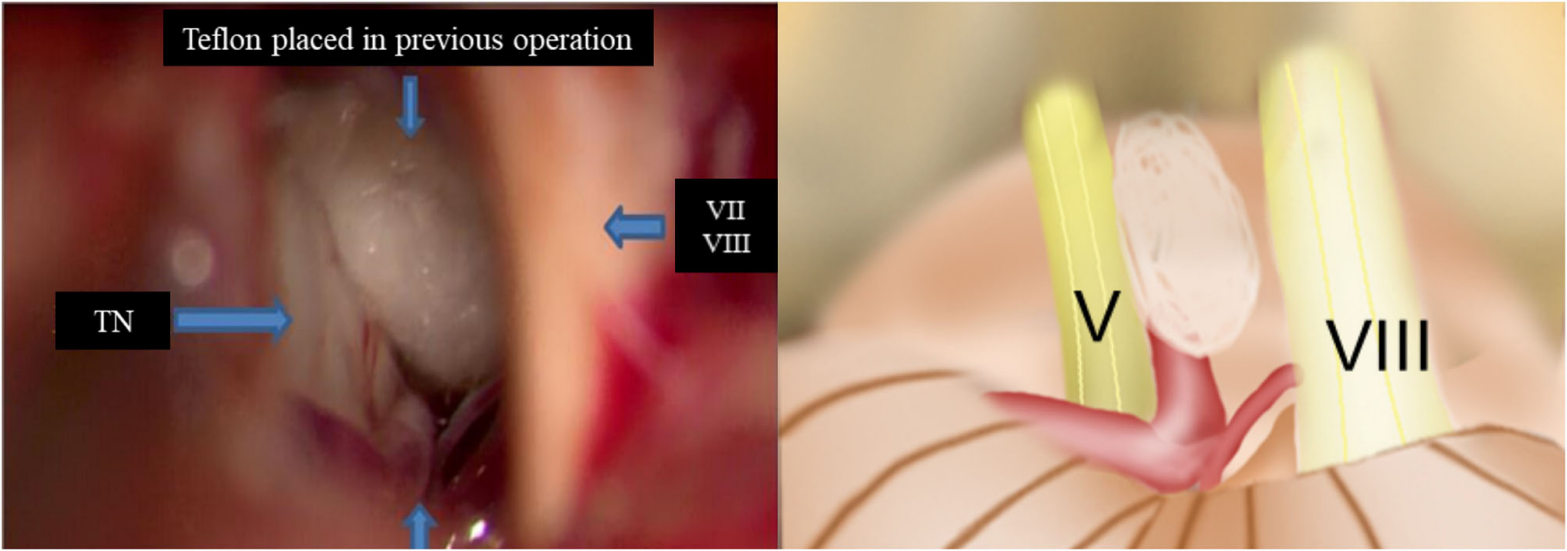
Intraoperative findings of redo MVD showing offender vessel omission in the previous operation. (TN, trigeminal nerve; VII, facial nerve; VIII, vestibulocochlear nerve).

**Figure 3:**
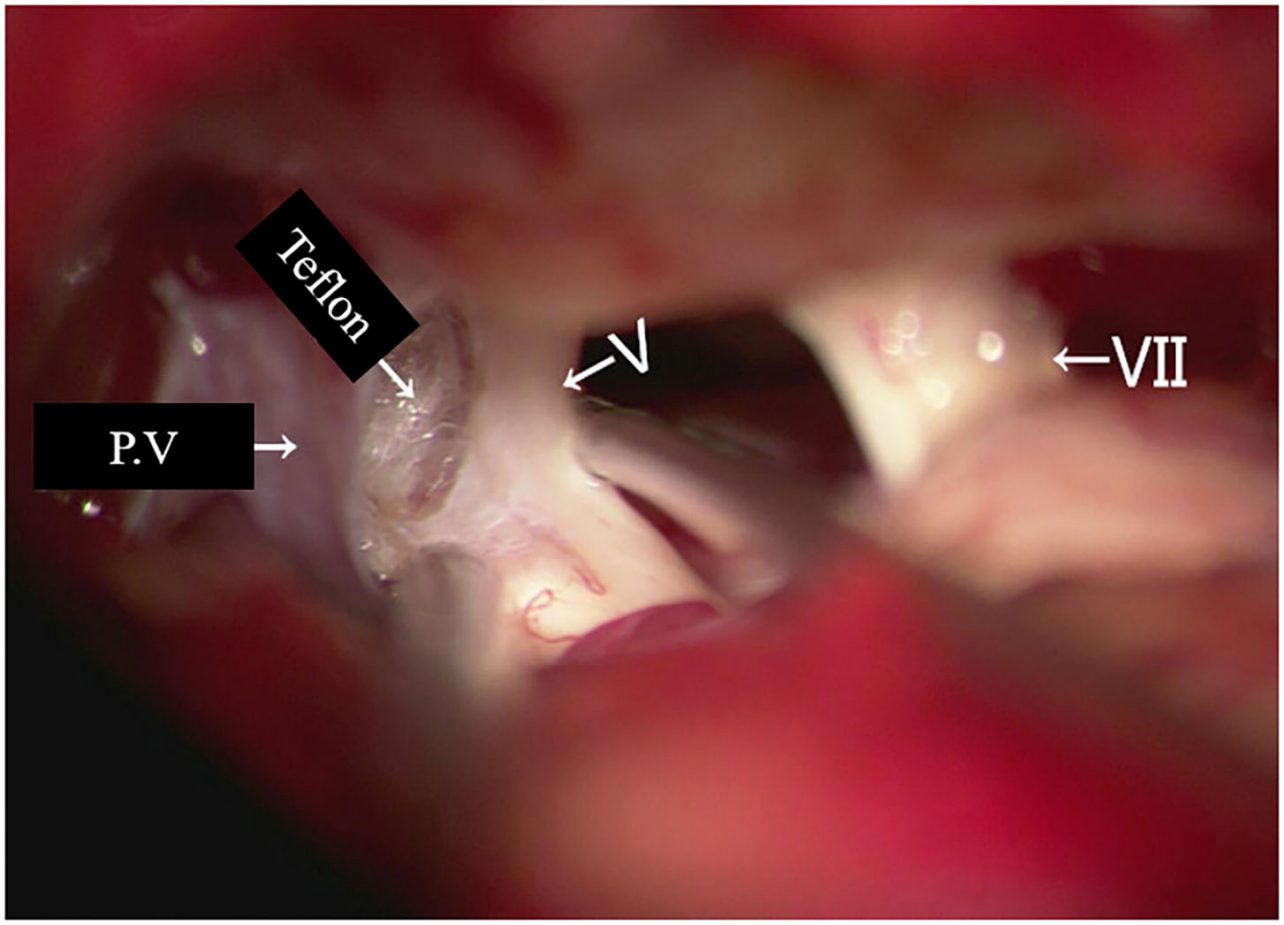
Teflon conflict with the nerve in redo MVD patients. (P.V, petrosal vein; V, trigeminal nerve; VII, facial nerve).

**Figure 4:**
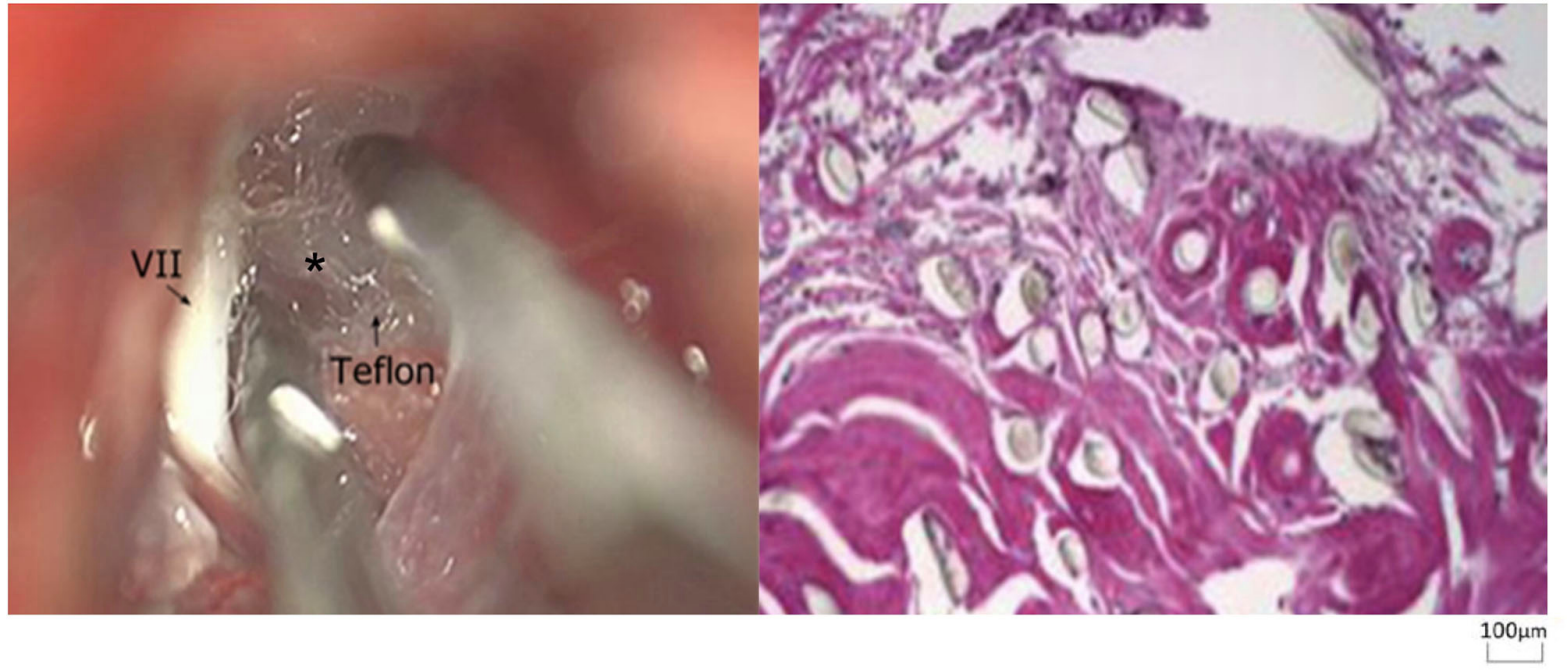
Teflon granuloma in redo MVD patients (hematoxylin and eosin staining, ×100). (VII, facial nerve; asterisk, granuloma).

Re-exploration involved reopening via the previous retrosigmoid approach. When clear evidence of vascular compression was found (Figure 2), a routine decompression procedure was carried out. If sponge or Teflon conflict with the nerve was suspected (Figure 3), the material was dissected and removed, the nerve was re-explored with all the material removed, and vascular decompression was carried out with new material. If the trigeminal nerve was distorted by the presence of Teflon granuloma (Figure 4), the nerve was freed via careful dissection. If multiple, complex blood vessels were compressed or if no vascular compression or trigeminal atrophy was noted, nerve combing was performed. The criteria for trigeminal atrophy were as follows: (A) the trigeminal nerve had become obviously thinner; and (B) compared with other nerves, the trigeminal nerve showed obvious denutrition and softening. For nerve combing, the trigeminal nerve was longitudinally divided along its fibers using a special nerve combing knife with a cutting edge of 0.90 mm into 3–5 bundles from the nerve root entry zone (REZ) to the petrous bone (5). To compare the therapeutic effects of nerve combing, we divided the patients with recurrence into a nerve combing group and a non-nerve combing group.

### Data Collection

Baseline data and medical history information were obtained from patient medical records. The baseline data included age at surgery, sex, the duration of symptoms, division of trigeminal nerve involved, the side of pain, operative findings, compression of the trigeminal nerve by vessels, postoperative pain relief, the presence of and time to recurrence, causes of recurrence and second operation outcomes. It should be noted that the age and duration at the first time the patients experimented the first episode of neuralgia, not the recurrence. Preoperative and postoperative statuses were based on visual analog scale (VAS) pain scores and Barrow Neurological Institute (BNI) pain intensity scores (6). VAS pain scores were recorded on a 11-point scale, with zero indicating no pain and 10 indicating maximum pain. The postoperative outcomes of TN were assessed with the BNI pain intensity score and the BNI facial numbness score, and the total of both scores was considered for further analysis (6) (Table 1). Patients were followed up at the outpatient department or by telephone. The data were collected prospectively using electronic operative records and case notes and retrospectively analyzed.

**Table 1:** Barrow Neurological Institute (BNI) pain intensity score, facial numbness score, and total evaluation of the results.

(P)Evaluation of pain relief by the BNI pain intensity score
1 No pain, no medication 2 Occasional pain, not requiring medication 3 Some pain, adequately controlled with medication 4 Some pain, not adequately controlled with medication 5 Severe pain/no pain relief
(N)Evaluation of numbness by the BNI facial numbness score
1 No facial numbness 2 Mild facial numbness, not bothersome 3 Facial numbness, somewhat bothersome 4 Facial numbness, very bothersome
(T) Total evaluation of results=(P) + (N)
2 Excellent 3 Good 4 Fair ≥5 Poor

### Statistical Analysis

SPSS statistical software 19.0 (IBM Corp., Armonk, NY, USA) was used for data analysis. Numerical variables are expressed as the mean ± SD. Qualitative variables are described as the absolute value of cases in the distinctive group. Statistical significance between the quantitative variables was assessed with the χ^2^ test and with Yates's or Fisher's correction if necessary. Student's *t*-test was performed to evaluate the data with a normal distribution. Repeated measure analysis of variance were used for statistical assessment. The survival data of different groups were determined using the Kaplan–Meier method and compared using the log-rank test (7). Univariable and multivariable Cox proportional hazards regression models were used to estimate the association of exclusion criteria and other clinically relevant prognostic factors. Variables assessed were Age, Gender, Side of Pain, duration of symptoms, Degree of pain (VAS), Division and Operative findings. Significant differences between groups were indicated when *p* < 0.05.

## Results

### Characteristics of the First MVD Procedure

A total of 1,900 consecutive TN patients underwent MVD from January 2004 to December 2015. A total of 127 recurrent TN patients (77 females and 50 males) were included in this study. Patient age at the time of the first surgery ranged from 28 to 89 years (mean 58.77 ± 14.51 years). The duration of pain before the first MVD procedure ranged from 0.5 to 10 years (mean 5.31 ± 2.99 years). The VAS scores before the first MVD procedure ranged from 7 to 10 (mean 9.27 ± 0.86). All patients had unilateral pain: 30 had pain on the left side, and 97 had pain on the right side. All three branches were affected in 23 of the 127 patients, two branches were affected in 48 patients, and one branch was affected in 56 patients. In 22 patients (13.32%), a large looped vertebrobasilar artery (VBA) was identified in the operative field and was regarded as the direct offending vessel compressing the trigeminal nerve. In 105 patients (82.67%), the offending vessels identified included single or multiple compressed vessels: the anterior inferior cerebellar artery (AICA), posterior inferior cerebellar artery (PICA), superior cerebellar artery (SCA), or petrosal veins without involvement of the VBA. The postoperative BNI scores after the first MVD procedure were excellent (T = 2) for 98 patients (77.17%), good (T = 3) for 22 patients (17.32%), and fair (T = 4) for 7 patients (5.51%).

We randomly selected 127 non-recurrent patients as the control group. A total of 82 females and 45 males were included, and the age of the participants ranged from 28 to 81 years (46.73 ± 12.94); among these patients, 44 were affected on the left side, and 83 were affected on the right side. The duration of TN ranged from 0.5 months to 10 years (2.69 ± 2.18 years). The preoperative VAS scores ranged from 7 to 10 (9.29 ± 0.87). All three branches were affected in 18 of the 127 patients, two branches were affected in 70 patients, and one branch was affected in 39 patients. Seven patients had VBA compression, and 120 patients had single or multiple compressed vessels. Overall, 120, 5, and 2 patients had excellent, good and fair postoperative BNI scores, respectively.

**Figure 5:**
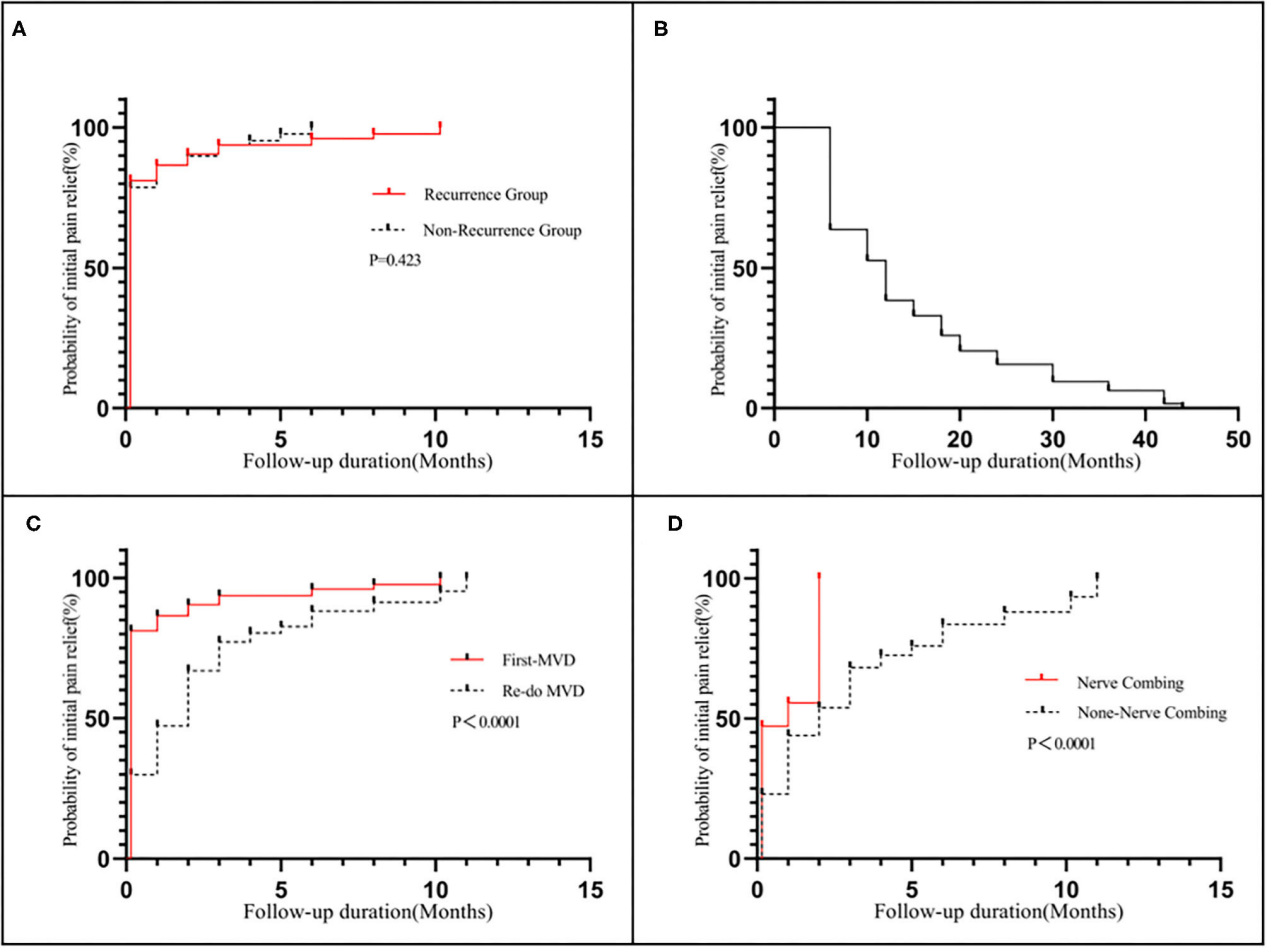
Kaplan–Meier analyses: **(A)** Revealed that although the non-recurrent group experienced initial pain relief earlier than the recurrent group after MVD, the results were not statistically significant (log-rank test, *p* = 0.423): **(B)** shows a median pain-free survival of 12 months after the first MVD procedure; **(C)** Revealed that the redo MVD group experienced initial pain relief later than the first-onset group after MVD, and the results were statistically significant (log-rank test, *p* < 0.05); **(D)** Revealed that the nerve combing group experienced initial pain relief earlier than the non-nerve combing group after MVD, and the results were statistically significant (log-rank test, *p* < 0.05).

The comparison between the recurrent and non-recurrent groups is summarized in Table 2. The age of the recurrent group was higher than that of the non-recurrent group (*p* < 0.05). The duration of pain before the first MVD procedure was longer in the recurrent group than in the non-recurrent group (*p* < 0.05). Patients in the recurrent group were more likely to have compression of the trigeminal nerve by the VBA or multiple vessels than patients in the non-recurrent group (*p* < 0.05). No significant differences were identified between the two groups in terms of sex, affected side, affected branches, preoperative VAS scores or postoperative BNI scores (*p* > 0.05). Kaplan–Meier analyses revealed that although the non-recurrent group experienced initial pain relief earlier than the recurrent group after MVD, the results were not statistically significant (Figure 5A; log-rank test, *p* = 0.423).

**Table 2:** Clinical characteristics of the recurrent and non-recurrent groups.

**Characteristic**	**Recurrence**	**Non-Recurrence**	***P***
Age	58.77 ± 14.51	46.73 ± 12.94	0.008
Sex: Female (%)	77 (60.63)	82 (64.57)	0.604
Side of Pain: Left (%)	30 (23.62)	44 (34.64)	0.550
duration of symptoms, years	5.31 ± 2.99	2.69 ± 2.18	<0.001
Degree of pain(VAS)	9.27 ± 0.859	9.29 ± 0.874	0.828
Division (%):			0.290
V1	6 (4.72)	3 (2.36)	
V2	17 (13.39)	13 (10.24)	
V3	33 (25.98)	23 (18.11)	
V1+V2	9 (7.09)	26 (20.47)	
V2+V3	39 (30.71)	44 (34.64)	
V1+V2+V3	23 (18.11)	18 (14.18)	
Operative findings (*n*):			<0.001
Single vessel compression	57 (44.88)	101 (79.53)	
Multiple vessel compression	48 (37.80)	19 (14.96)	
Vertebrobasilar artery compression	22 (13.32)	7 (5.51)	
BNI grade (*n*)			0.130
II	98 (77.17)	120 (94.49)	
III	22 (17.32)	5 (3.94)	
IV	7 (5.51)	2 (1.57)	

### Characteristics of Redo MVD

**Figure 6:**
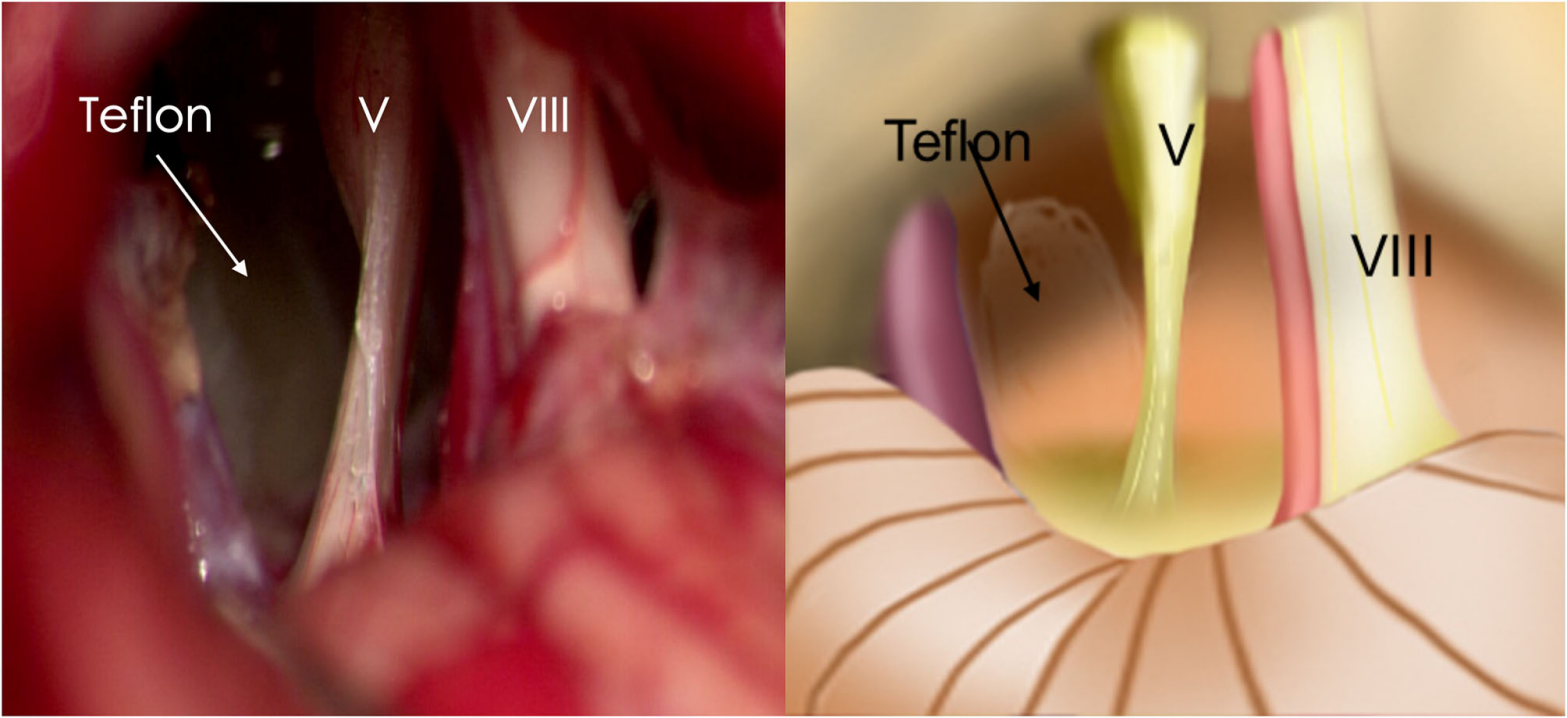
Trigeminal atrophy was observed during MVD. (V, trigeminal nerve; VIII, vestibulocochlear nerve).

The time to pain recurrence after the first MVD procedure was 6 to 44 months (15.02 ± 10.76 months). The Kaplan–Meier curve showed a median pain-free survival of 12 months after the first MVD procedure (Figure 5B). All three branches were affected in 21 (16.50%) of the 127 patients, two branches were affected in 57 patients (44.88%), and one branch was affected in 49 patients (38.62%). The preoperative VAS scores ranged from 7 to 10 (8.96±0.97). Among all 127 patients, single-vessel compression was found in 7 (5.51%) patients, and multiple-vessel compression was found in 11 (8.66%) patients. VBM compression was found in 12 (9.45%) patients. Teflon compression or granuloma was found in 41 (32.28%) and 23 (18.11%) patients, respectively. The remaining 33 (25.99%) patients had no vascular compression, which was considered the main cause of trigeminal atrophy (Figure 6). The postoperative BNI scores after the first MVD procedure were excellent (*T* = 2) for 69 patients (53.33%), good (*T* = 3) for 46 patients (36.22%) and fair (*T* = 4) for 13 patients (10.48%). A total of 80 (62.99%) patients suffered from facial numbness postoperatively. Two (1.57%) patients suffered from hearing loss. Ten (7.87%) patients and 5 (3.93%) patients suffered from CSF leakage and wound infection, respectively. Eight (6.32%) patients had transient blurred vision. During the follow-up, no recurrence was noted. All complications were cured or improved. The postoperative follow-up was 63 to 167 months (105.92 ± 25.66).

The comparison between the first and redo MVD groups among recurrent patients is summarized in Table 3. No significant difference was identified between the two groups in terms of affected branches (*p* > 0.05). However, the severity of pain (preoperative VAS score) in patients with recurrence was lower than that in patients with first-onset TN (*p* < 0.05). The postoperative BNI scores for patients with recurrence were higher than those for patients with first-onset TN (*p* < 0.05). Kaplan–Meier analyses revealed that the redo MVD group experienced initial pain relief later than the first-onset group after MVD, and the results were statistically significant (Figure 5C; log-rank test, *p* < 0.05).

**Table 3:** Clinical characteristics of the first and re-do MVD groups in recurrent patients.

**Characteristic**	**first MVD**	**re-do MVD**	***P***
Degree of pain(VAS)	9.27 ± 0.86	8.96 ± 0.97	0.001
Division (%):			0.438
V1	6 (4.72)	9 (7.08)	
V2	17 (13.39)	13 (10.24)	
V3	33 (25.98)	27 (21.30)	
V1+V2	9 (7.09)	18 (20.47)	
V2+V3	39 (30.71)	39 (24.41)	
V1+V2+V3	23(18.11)	21 (16.50)	
BNI grade (*n*)			0.000
II	98 (77.17)	69 (53.33)	
III	22 (17.32)	46 (36.22)	
IV	7 (5.51)	13 (10.45)	

If multiple, complex blood vessels were compressed or if no vascular compression or trigeminal atrophy was noted, nerve combing was performed. In this study, 36 (28.34%) recurrent patients were treated with nerve combing. The comparison between nerve combing and non-nerve combing groups among recurrent patients is summarized in Table 4. No significant differences were found between the two groups in terms of postoperative BNI scores or complications (*p* > 0.05). Kaplan–Meier analyses revealed that the nerve combing group experienced initial pain relief earlier than the non-nerve combing group after MVD, and the results were statistically significant (Figure 5D; log-rank test, *p* < 0.05).

**Table 4:** Clinical characteristics of the nerve combing and non-nerve combing groups in recurrent patients.

**Characteristic**	**Non-Nerve Combing**	**Nerve Combing**	***P***
	**(*n* = 91)**	**(*n* = 36)**	
Complications (*n*)			0.130
None	20 (21.98)	2 (5.56)	
Facial numbness	54 (59.34)	26 (72.22)	
Hearing loss	2 (2.20)	0 (0)	
CSF leakage	5 (5.49)	5 (13.89)	
Wound infection	4 (4.40)	8 (2.78)	
Blurred vision	6 (6.59)	2 (5.55)	
BNI grade(n)			0.260
II	44 (48.35)	25 (69.44)	
III	34 (37.36)	12 (30.56)	
IV	13 (14.29)	0	

## Discussion

### Clinical Features of Recurrent Trigeminal Neuralgia

According to the classification and diagnostic grading of TN issued by the International Association for the Study of Pain (IASP), TN is distinguished into three diagnostic categories: typical and atypical (secondary and idiopathic) (8). Typical TN has four characteristics: (1) recurrence of paroxysmal attacks lasting from a fraction of a second to 2 min, (2) severe intensity, (3) electric shock-like, shooting, stabbing or sharp in quality, and (4) precipitated by innocuous stimuli on the affected side of the face (9). Atypical TN usually involves pain lasting more than a few minutes or burning pain (10). The etiology of typical TN is not completely clear, and most studies suggest that microvascular compression of the nerve root is the main cause of typical TN (11). Based on the above theoretical basis, MVD for the treatment of primary TN has been increasingly applied in clinical practice (12). The clinical treatment of primary TN with MVD surgery has a cure rate of 80–98%, but the follow-up recurrence rate is 10–30% (3). In this study, 11.05% of the patients experienced TN recurrence, which is lower than the rates reported in previous studies (13–15). We believe that the key to preventing recurrence is to accurately diagnose the type of TN before MVD. In addition to differences in clinical manifestations, magnetic resonance imaging technology, such as 3D T1- and T2-weighted high-resolution sequences and 3D time-of-flight MRA, can not only accurately show the neurovascular compression area and offending vessels but also diagnose TN alongside multiple sclerosis, a rare cause of the risk factors for atypical TN (16).

Patients with recurrent TN show some clinical features. Previous studies have shown that 2% to 3.5% of cases of recurrence occur within 1 year, whereas most cases of recurrence occur within 2 years (17). Mendoza (18) reported that patients who experienced recurrence within 2 years after MVD accounted for 90% of recurrent cases. Our results are similar to those of previous studies. In our experience, the clinical manifestations of patients with recurrence are often different from those before the first MVD procedure. These patients tend to exhibit the following three characteristics: 1, pain changing from typical before the first operation to atypical (e.g., poor drug control); 2, decreased sensation on the affected side of the face; and 3, a changing area of pain from onset in some patients (Table 3).

### Trigeminal Nerve Atrophy

Patients with trigeminal nerve atrophy had a higher pain recurrence rate after experiencing initial pain relief than those without trigeminal nerve atrophy (19). In our study, 25.99% of the patients had trigeminal atrophy (Figure 6). Hilton et al. (20) observed that the myelin sheath of the affected trigeminal nerve was degenerated, the myelin-sheathed axons were close together and arranged in parallel, without inflammatory cell infiltration, and astrocytes could be observed around the lesion. The trigeminal nerve changes induced by multiple sclerosis are similar to those described above but may be accompanied by inflammatory cell infiltration and glial hyperplasia. Since no pathological studies were conducted in this report, further studies are needed to verify the etiology of trigeminal atrophy. Both neurodegeneration and multiple sclerosis are closely related to age (21), which is consistent with the higher recurrence rate among the elderly patients than that among the younger patients in our study.

However, a systematic review (22) found that the elderly population had lower recurrence rates, which may be related to the presence of cerebral atrophy in elderly patients, resulting in clear exposure of the intraoperative cerebellar pontine angle (CPA) area and thus facilitating separation of the responsible vessels from the trigeminal nerve (23, 24). We believe that with age, atherosclerosis and other age-related changes in the body weaken the function of the nerve-humoral barrier, reducing the adaptive and compensatory responses of the nervous system. Sabalys et al. (25) proposed that vascular compression or the anaphylaxis-immune response results in trigeminal dystrophy, a key prerequisite for initiation of the pathogenesis of TN. Aging of the body is more conducive to the pathogenesis of TN, which may explain why elderly patients are more likely to experience recurrence. In this study, our results also showed that the duration of pain before the first MVD procedure was longer in the recurrent group, possibly because prolonged compression causes REZ damage and more severe demyelination of the trigeminal nerve (26), increasing the likelihood of recurrence.

For trigeminal nerve atrophy patients, nerve combing is recommended based on our experience, which involves longitudinal combing of the sensory roots of the trigeminal nerve with a special knife (5). In contrast to neurotomy, combing maintains the integrity of nerve roots and maximizes the function of nerve fibers. Postoperative facial numbness is mild, and the operation is safe (27). In this study, the nerve combing group experienced initial pain relief even earlier than the non-nerve combing group after MVD (Figure 5D), suggesting that nerve combing treatments offer a good outcome for recurrent patients with trigeminal nerve atrophy (28).

### Incomplete Decompression or Omission of Responsible Vessels

The recurrence rate after MVD is closely related to the surgical methods and whether decompression of responsible vessels is complete. In our study, 30 of 127 patients had recurrence due to vascular compression, which was caused by Teflon displacement, insufficient or omitted decompression of offending vessels or new responsible vessels. Incomplete decompression or omission of responsible vessels is one of the most important reasons for recurrence (29).

Anatomically, the trigeminal nerve, as the largest cranial nerve, is divided into three parts, and the part located in the cisterna before entering the pons is called the REZ, which is the most vulnerable to vascular compression (30); thus, the REZ should be fully exposed during the operation (31). However, only probing and separating the responsible vessels in the REZ are insufficient, and responsible vessels in other parts or unknown sources of pain must be carefully explored (32). The unmyelinated part of the intracranial segment of the trigeminal nerve is longer, and its resistance to peripheral vascular compression is poor. Nerve vascular compression may occur in any part of the nerve root, and any vessel in anatomical contact with the posterior root of the trigeminal nerve may be a responsible vessel, such as the petrosal vein and its branches (33). The entire intracranial segment of the nerve root should be exposed during MVD.

In addition, using the original incision is generally recommended for reoperation (34). Therefore, we believe that the design of the first MVD surgical incision and full exposure of the trigeminal nerve have an important influence on the recurrence of TN. Furthermore, good surgical habits during surgery can also ensure a low recurrence rate, for example, following the partition order to avoid omissions. In our previous study, the surgical area was divided by the entry zone of the trigeminal nerve root into 5 areas: the dorsal quadrant, the caudal quadrant, the head lateral quadrant, the ventral quadrant and the trigeminal nerve trunk (35). During MVD, these regions should be detected in sequence to effectively avoid omission of responsible blood vessels.

Notably, a large looped VBA was identified as the direct offending vessel in 12 patients. Some authors have suggested using the “stitched sling retraction” technique, where the VBA can be pushed away from the REZ region while minimizing contact with the trigeminal nerve as much as possible (36, 37). However, such manipulation might require a wider working space and may endanger vessels (38). For large offending arteries, such as the VBA, our team never used the vessel transposition technique or medical adhesive products. Small pieces of wet gelatin sponge were inserted first to expand the gap, and then Teflon felt was placed for interposition, which is recommended (38).

### Teflon Granuloma

Teflon granuloma was also a reason for recurrence (Figure 4). According to previous studies (39, 40), the incidence of TF is ~1.1–7.3%. A Teflon granuloma forms as a result of an inflammatory reaction to polytetrafluoroethylene fibers, which may be triggered by Teflon contact with blood or the dura (40, 41). In this study, 23 patients had recurrence due to Teflon granulomas, which is higher than the number reported in the literature and might be related to the application of Teflon (41). To avoid the formation of a Teflon granuloma, we implemented the following two procedures: 1, we pushed the responsible blood vessel as far away as possible from the trigeminal nerve with Teflon; that is, we did not touch or compress the trigeminal nerve with a cotton pad; and 2, we did not leave any blood on the Teflon. The pain associated with Teflon granuloma-induced recurrent TN often involves new TN branches and is accompanied by facial hypoesthesia (40, 41). MRI enables diagnosis of a granuloma, which appears as a low-to-intermediate-signal intensity lesion on T1/T2 sequences. Surgical removal of granuloma combined with redo MVD is the preferred treatment.

### Complications

In addition to hearing loss, CSF leakage and wound infection, 80 (62.99%) patients suffered from facial numbness postoperatively in this study. We believe that the following measures can help prevent the occurrence of facial numbness: (1) avoiding disturbance or stretching of the trigeminal nerve; (2) avoiding electrocoagulation of the nerve surface as electrocoagulation can lead to paroxysmal episodes of numbness; (3) ensuring that the Teflon sponge does not conflict with the nerve; and (4) avoiding cutting part of the nerve; the combing technique can be used to reduce denervation symptoms. In addition, 8 patients had transient blurred vision, which may be related to mild keratitis caused by damage to the ophthalmic nerve, the first branch of the trigeminal nerve (42–44). Most sensory nerve fibers of the cornea, conjunctiva and accessory organs of the eye originate from the ophthalmic nerve, and many nutrient factors secreted by the ophthalmic nerve play an important role in maintaining the integrity of the structure and function of the eye surface, which can maintain homeostasis of the eye surface, regulate metabolism and promote wound healing (45). In summary, for recurrent patients, redo MVD can yield good results. However, performing percutaneous lesioning surgery, especially in patients in whom post-operative MRI does not show evidence of strong remaining root compression, may be justified. As a matter of fact, redoing MVD may result in neural trauma linked to dissection/manipulation of the root. Indeed, as shown in Table 4, 59% of the patients without combing and 72% of the patients with combing experienced facial numbness, indicating a deficit in nerve conduction. The same result may have likely been obtained by less invasive methods.

However, the postoperative BNI scores of the recurrent patients were higher than those of the patients with first-onset TN (*p* < 0.05). Kaplan–Meier analyses also revealed that the redo MVD group experienced initial pain relief later than the first-onset group after MVD (Figure 5C), indicating that redo MVD is always less effective than initial MVD. Therefore, neurosurgeons should focus more on the treatment of patients with recurrence. We believe that with accumulation of surgical experience and improvement of operating skills, patient satisfaction can be improved.

## Conclusions

Microvascular decompression (MVD) is an effective surgical method for the treatment of TN. For recurrent patients, reoperation can yield good results. In summary, we studied the clinical characteristics of and surgical treatment strategies for recurrent TN, providing guidance for clinicians in the diagnosis and treatment of this disease.

## Data Availability Statement

The raw data supporting the conclusions of this article will be made available by the authors, without undue reservation.

## Ethics Statement

The studies involving human participants were reviewed and approved by the institutional review board of the Jiangxi provincial People's Hospital, the Seventh Medical Center of PLA General Hospital and Characteristic Medical Center of Strategic Support Force. The patients/participants provided their written informed consent to participate in this study. The study participants provided their consent for publication of any data/associated images.

## Author Contributions

JL and RL contributed to the writing of this manuscript. GW, HX, FL, BH, WQ, HS, and ZL contributed to the study conception and design and editing of the manuscript.

## Conflict of Interest

The authors declare that the research was conducted in the absence of any commercial or financial relationships that could be construed as a potential conflict of interest.
